# Novel transcriptional signatures for sputum-independent diagnostics of tuberculosis in children

**DOI:** 10.1038/s41598-017-05057-x

**Published:** 2017-07-19

**Authors:** John Espen Gjøen, Synne Jenum, Dhanasekaran Sivakumaran, Aparna Mukherjee, Ragini Macaden, Sushil K. Kabra, Rakesh Lodha, Tom H. M. Ottenhoff, Marielle C. Haks, Timothy Mark Doherty, Christian Ritz, Harleen M. S. Grewal

**Affiliations:** 1 Department of Clinical Science, Faculty of Medicine, University of Bergen, Bergen, Norway; 2 Department of Infectious Diseases, Oslo University Hospital, Oslo, Norway; 3 Department of Pediatrics, All India Institute of Medical Sciences, New Delhi, India; 4 Division of Infectious Diseases, St. John’s Research Institute, Koramangala, Bangalore India; 5 Department of Infectious Diseases Group, Immunology and Immunogenetics of Bacterial Infectious Disease, Leiden University Medical Center, Leiden, The Netherlands; 6 GlaxoSmithKline Vaccines, Wavre, Belgium; 7 Department of Nutrition, Exercise and Sports, University of Copenhagen, Copenhagen, Denmark; 8 Department of Microbiology, Haukeland University Hospital, University of Bergen, Bergen, Norway

## Abstract

Pediatric tuberculosis (TB) is challenging to diagnose, confirmed by growth of *Mycobacterium tuberculosis* at best in 40% of cases. The WHO has assigned high priority to the development of non-sputum diagnostic tools. We therefore sought to identify transcriptional signatures in whole blood of Indian children, capable of discriminating intra-thoracic TB disease from other symptomatic illnesses. We investigated the expression of 198 genes in a training set, comprising 47 TB cases (19 definite/28 probable) and 36 asymptomatic household controls, and identified a 7- and a 10-transcript signature, both including *NOD2*, *GBP5*, *IFITM1/3*, *KIF1B* and *TNIP1*. The discriminatory abilities of the signatures were evaluated in a test set comprising 24 TB cases (17 definite/7 probable) and 26 symptomatic non-TB cases. In separating TB-cases from symptomatic non-TB cases, both signatures provided an AUC of 0.94 (95%CI, 0.88–1.00), a sensitivity of 91.7% (95%CI, 71.5–98.5) regardless of culture status, and 100% sensitivity for definite TB. The 7-transcript signature provided a specificity of 80.8% (95%CI, 60.0–92.7), and the 10-transcript signature a specificity of 88.5% (95%CI, 68.7–96.9%). Although warranting exploration and validation in other populations, our findings are promising and potentially relevant for future non-sputum based POC diagnostic tools for pediatric TB.

## Introduction

Tuberculosis (TB) ranks as a leading cause of childhood death and morbidity worldwide, estimated to cause 1 million new cases yearly in children <15 years^1^. In fighting the epidemic, it is of great concern that the detection rate for pediatric TB is only 35%^2^.

The gold standard for a diagnosis of pulmonary TB is growth of *Mycobacterium tuberculosis* (*Mtb*) from respiratory specimens. Since 2010, the use of the Xpert MTB/RIF on sputum samples has been implemented rapidly in low- and middle income countries^3^, serving as a point-of-care test (POC-test) with improved diagnostic accuracy in adults compared to direct microscopy^1^. However, in children, the Xpert MTB/RIF has clear limitations because of the paucibacillary nature of disease and the difficulties in obtaining representative specimens^4^. The majority of pediatric TB cases are therefore diagnosed through clinical scoring systems with obvious shortcomings^5, 6^ related to the nonspecific nature of signs, symptoms and radiological findings^7^, and growing evidence for reduced sensitivity of the interferon-gamma release essays (IGRAs) and the tuberculin skin test (TST) in young and malnourished children^8–10^. Although over-diagnosis can occur, under-diagnosis is more common, contributing to morbidity, death and masking the true burden of pediatric TB^6^.

Little attention was paid to pediatric TB by public health authorities until WHO declared pediatric TB a neglected area and called for research to address the lack of adequate diagnostics for children^11^, encouraging in particular biomarker research to fill this gap^12^, preferably differentiating children with TB from symptomatic non-TB cases^13^. The search for TB biomarkers based on analyses of human gene expression has received increasing attention, but data in children remains limited^14, 15^. A landmark study by Anderson *et al*.^14^ using genome-wide analysis of RNA expression in whole blood (WB) in three cohorts of African children discovered a 51-transcript signature from which they derived a risk-score distinguishing TB from other diseases. Similarly, Verhagen *et al*.^15^ found a 116-gene signature and identified a 5-gene set that discriminated TB disease from non-TB pneumonia in Warao-Amerindian children. The findings from these genome-wide analyses were remarkably different; no genes overlapped between the 51- and the 116-transcripts. In the search for consistency in human gene expression related to TB pathology across age groups and national borders, Sweeney *et al*. applied gene expression data from publically available microarray repositories, using three discovery datasets from adult TB to identify a 3-gene combination, that separated TB from other diseases in two datasets from children, and in one dataset from adults, with a mean AUC of 0.83 for the three cohorts^16^.

Studies based on genome-wide analyses of transcriptomes are expensive and extremely resource-demanding and tend to generate large biomarker signatures (which might be difficult to reduce to clinically practical tests), but represent important steps towards a POC-test for TB, adding novel, un-biased information of expression of genes with relevance to TB risk and pathogenesis^17–23^. We have previously explored the expression and differential capacity of a pre-selected panel of host transcripts with possible involvement in TB pathogenesis in Indian children^24, 25^, but have continued to expand the gene panels as novel evidence accumulates. In the present study, we have incorporated type I IFN-inducible genes and a broader panel of genes covering general inflammation, myeloid cell activation and humoral immunity, in a user-friendly and inexpensive technique; the dual-color-Reverse-Transcriptase-Multiplex-Ligation-dependent-Probe-Amplification (dc-RT MLPA). This technique has excellent abilities for profiling host biomarkers in larger sample sets, with a dynamic range and accuracy comparable to qPCR, requiring small amounts of RNA per sample^26^. Based on the knowledge gained from multiple genome-wide analyses, we aimed to find more defined transcriptional signatures in unstimulated WB of Indian children, with the ability to separate TB cases from young children symptomatic for other reasons, resembling a real-life diagnostic setting in India, the country carrying the greatest share of the global TB burden^1^.

## Methods

### Source population

**Figure 1:**
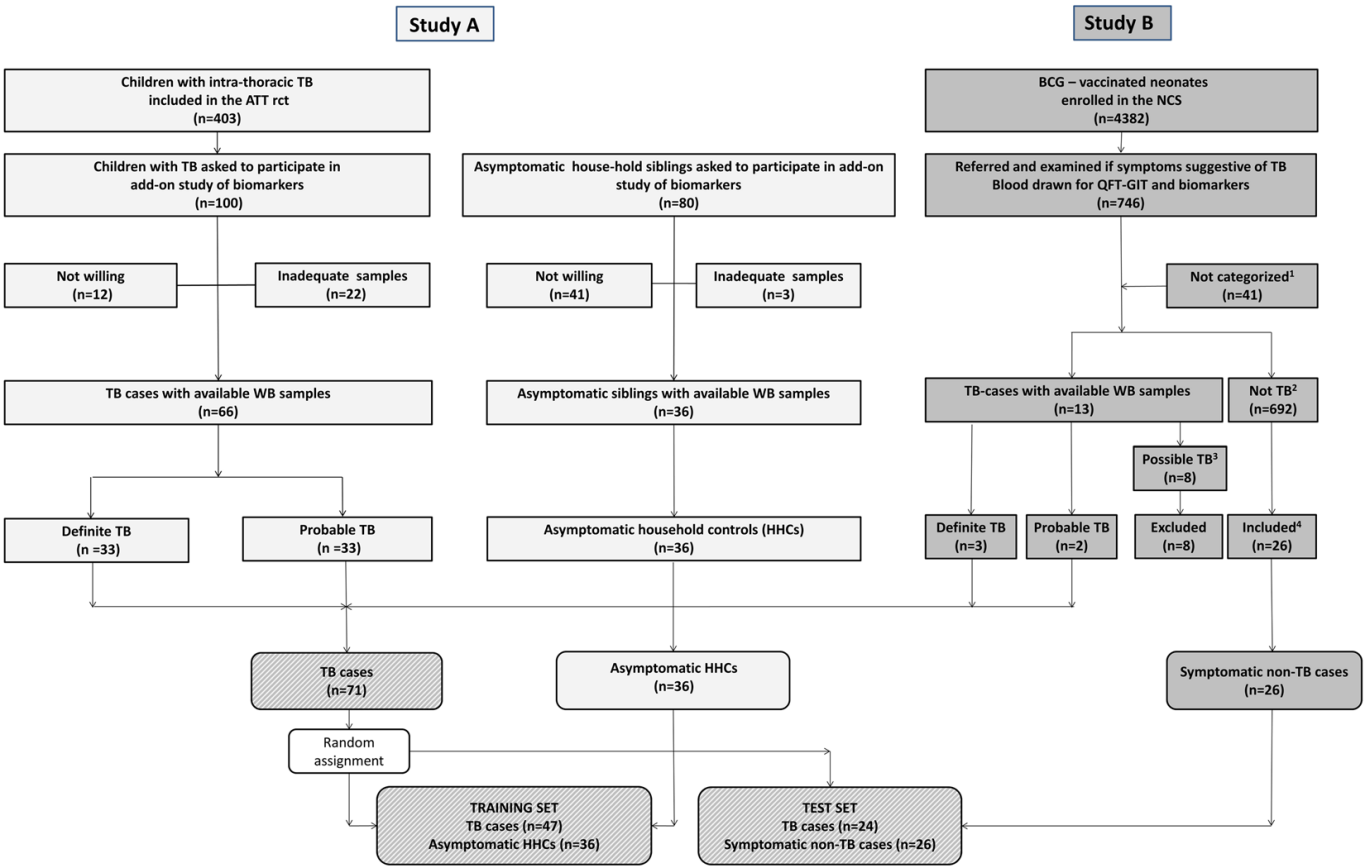
Study flowchart. Selection of participants from study A (Light grey boxes) and study B (Darker grey boxes). Hatched grey boxes: Participants from both studies. Study A was a randomized-controlled trial (rct) of the effect of different micronutrient supplementary as an adjunct to anti-tuberculosis therapy (ATT), carried out from January 2008 to June 2012 in Delhi, India. Study B: A neonatal cohort study (NCS) of BCG-vaccinated neonates randomized to active or passive surveillance for 3 years, in Palamaner Taluk, India, April 2007 to September 2010. ^1^Inadequate samples or lost to follow-up. ^2^Ninety of 692 were either QFT/TST positive, or both, indicating *M. tuberculosis (Mtb)* infection. ^3^Criteria for possible TB: ≥1 sign and symptom for TB, and either; response to treatment/documented exposure/immunological evidence of *Mtb*-infection, or; X-ray consistent with TB. ^4^TB ruled out by clinical, radiological and microbiological examination.

This study is cross-sectional and draws on WB samples from two prospective clinical studies (A and B) previously conducted in India (Fig. 1). Briefly, study A was a randomized controlled trial of the effect of micronutrient supplementation as an adjunct to anti-tuberculosis therapy in children diagnosed with intra-thoracic TB (Delhi) from January 2008 to June 2012^27^. Study B was a prospective study of BCG-vaccinated neonates, randomized to 2-year active or passive surveillance (Palamaner Taluk, Andhra Pradesh) from April 2007 to September 2010^9^.

### Referral criteria study A and B

*Study A;* children aged 6 months to 15 years were screened for TB at admittance to a tertiary hospital if either; cough and/or fever ≥2 weeks with no improvement after 7–10 days of amoxicillin; recent unexplained weight loss/failure to thrive (FTT); fatigue/lethargy or subtle clinical symptoms and close contact with an adult with TB. Household siblings of the included TB cases were investigated for concomitant TB disease.

*Study B;* children <3 years were referred to a case verification ward if either; >8 hour exposure to a TB patient within the last year; cough and/or fever ≥2 weeks; FTT or a TST ≥ 10 mm at study closeout.

### Diagnostic assessment study A and B

A medical history and clinical, demographic and anthropometric data were recorded. A TST was performed by a trained nurse/doctor (induration of ≥10 mm was defined positive), and peripheral WB was drawn for the QuantiFERON®-TB Gold In-Tube (QFT) (Cellestis, Australia), performed per the manufacturer’s instructions. Chest X-rays (CXR) were interpreted by 3 independent radiologists requiring agreement by 2 for a diagnosis of TB disease. Gastric aspirates and induced sputa were collected on 2 consecutive days for direct Ziehl-Neelsen (Study A) or fluorescent microscopy (Study B), and culture.

For asymptomatic siblings, a medical history and clinical, demographic and anthropometric data were recorded, and a TST and a CXR performed.

The prevalence of HIV infection was assessed anonymously (study A) or extracted (study B) from a household contact study in the same area (TB trials study group, unpublished data), and found to be <1% in both settings.

### Selection and classification of participants for the present study

#### TB cases

Samples from children with intra-thoracic TB disease were selected from study A and B. According to consensus guidelines^5^, children were categorized as having either definite TB (*Mtb* confirmed in ≥1 culture and ≥1 sign/symptom suggestive of TB, e.g. non-remitting cough >2 weeks; unexplained fever >1 week; weight loss; FTT), or probable TB (CXR consistent with TB, ≥1 sign/symptom and documented TB exposure or immunological evidence of *Mtb*-infection).

#### Asymptomatic household controls (asymptomatic HHCs)

Samples from siblings of TB cases were selected from study A.

#### Symptomatic controls without TB (symptomatic non-TB cases)

Children having ≥1 sign/symptom, in whom TB disease was excluded by diagnostic work-up, were selected from study B.

### Selection of transcriptional biomarkers

A total of 198 genes (including 4 housekeeping genes), distributed in 3 panels, were selected for use in dc-RT MLPA. Thirty genes were present in more than one panel. Genes and gene names for the 145 unique genes are given in Supplementary Table S1.

The first 48-gene set has been described in our previous studies^24, 25^. The second 92-gene set included genes known for involvement in general inflammation and myeloid cell activation, and genes involved in the adaptive immune system, comprising Th1/Th2-responses, regulatory T-cell markers and B-cell associated genes^28^. The third 58-gene set included type 1- interferon inducible genes^17^ known to be up-regulated in adult TB disease, and genes associated with predicted risk for TB disease in South African neonates^29^, to explore their diagnostic potential in paediatric TB.

### Sample collection and RNA-extraction

Peripheral WB (1.0–2.5 ml) was drawn into PAXgene blood RNA tubes (PreAnalytiX, Hombrechtikon, Switzerland) and stored at −80 °C until RNA extraction (PAXgene Blood RNA kit; PreAnalytiX, Hilden, Germany). Total RNA concentration and purity were measured using a Nanodrop spectrophotometer (Thermoscientific, Wilmington, DE, USA) and ranged between 0.4–24.5 µg (average 6.6 ± 4.85 µg).

### dual-color-Reverse-Transcriptase-Multiplex-Ligation-dependent-Probe-Amplification (dcRT-MLPA)

For each target sequence, a specific RT primer was designed, located immediately downstream of the left and right hand half-probe target sequence. A total RNA of 125 ng was used for reverse transcription, applying MMLV reverse transcriptase (Promega, Madison, WI, USA), followed by hybridization of left and right hand half-probes to the cDNA at 60 °C overnight. Annealed half-probes were ligated and the ligated product was subsequently amplified by PCR. The remaining steps were performed as described elsewhere^30^. All 133 samples were run in two (96-well) plates for each of the gene panels. The PCR fragments were analysed on a 3730 capillary sequencer in Gene scan mode (Life Technologies, Carlsbad, CA, USA), using GeneMapper version 5.0 (Life Technologies, Carlsbad, California, USA). Primers and probes were obtained from the Department of Infectious Diseases, Leiden Medical University, the Netherlands.

### Statistical analysis

TB cases from study A and B were randomly divided to 2/3 in a training set, and 1/3 in a test set. This design was adopted to counterbalance the fact there were relatively few TB cases in study B compared to study A. Asymptomatic HHCs from study A constituted the controls of the training set, whereas symptomatic non-TB cases from study B constituted the controls of the test set (Fig. 1, Table 1).

**Table 1:** Clinical characteristics of study subjects and distribution to training and test set.

	Training set			Test set		
	TB disease n = 47 (%)	HHCs n = 36 (%)	p-value	TB disease n = 24 (%)	Non-TB cases n = 26 (%)	p-value
	Definite = 19 (40)			Definite = 17 (70)		
	Probable = 28 (60)			Probable = 7 (30)		
**Demographics**						
Age in months (mean)	108	104	0.47	102	19	<0.0001
Range	9–175	12–216		24–175	2–27	
Gender (male)	19 (40)	19 (53)	0.26	11 (46)	18 (69)	0.09
**Mycobacterial exposure**						
Known BCG vaccination	41 (87)	28 (78)	0.25	23 (96)	26 (100)	0.29
Known TB exposure	16 (34)	36 (100)	<0.0001	8 (33)	4 (15)	0.14
**Tuberculin skin test**						
Positive (≥10 mm)	44 (94)	15 (42)	<0.0001	24 (100)	10 (38)	<0.0001
Median (mm)	18	15		19	6	
**Quantiferon Gold in tube**						
Positive (≥0,35 IU/mL)	31 (66)	NA^3^		17 (71)	9 (35)	0.01
Indeterminate	1 (2)	NA^3^		0	0	
Median (IU/mL)	1.6	NA^3^		1.5	0.035	
**Symptoms**						
Cough >2 weeks	28 (60)	0	<0.0001	16 (67)	13 (50)^4^	0.23
Fever >1 week	38 (81)	0	<0.0001	17 (71)	9 (35)^4^	0.01
Weight loss/Failure to thrive^1^	35 (75)	1 (3)	<0.0001	18 (75)	23 (88)	0.94
**Findings**						
Abnormal Chest X-ray	46(98)	0	<0.0001	23 (96)	1 (4)	<0.0001
BMI-for-age <−2 Z-Scores^2^	21 (45)	9 (25)	0.05	14 (58)	15 (58)	0.96

^1^Definition “Failure to thrive”: Loss of weight or no weight gain for 2 consecutive visits; downward crossing of 2 percentile lines on the weight-for-age growth chart, or weight that tracked consistently below the 3rd percentile in the weight-for-age growth chart.

^2^Body Mass Index-for-age <2 Z-scores defined as thinness according to WHO.

^3^QFT not undertaken for asymptomatic controls.

^4^No criteria for length of symptoms for the symptomatic non-TB cases in the present study.

We used the training set to identify transcriptional signatures from the dc-RT MLPA data, by applying two different statistical learning approaches that relied solely on cross validation as a means for gauging predictive power of genes, both individual genes and combined, i.e.; no statistical significance tests were used.

In the first statistical approach, a transcriptional signature was obtained by applying LASSO^31^ (Least absolute shrinkage and selection operator) regression analysis directly on the dc-RT MLPA data for expression levels from all unique genes. For repeated genes (30), the panel that represented the highest mean expression value for the biomarker was used. LASSO analyses included adjustment for age, i.e. age was included as a covariate in all steps of the analyses. Optimal tuning parameters were found using a cross validation step, which was repeated 100 times to stabilize results.

In the second approach, to eliminate the risk of overfitting due to the large number of genes, we performed an initial filtering of all biomarkers, using cross validation based on logistic regression^31^ on gene expression data for each biomarker separately, retaining only biomarkers with a cross-validated prediction ≥0.7. Then LASSO analysis was applied on the reduced collection of retained biomarkers to identify a transcriptional signature.

The two signatures obtained were then evaluated in the test set, without any retrospective optimization. Lasso weights based on the analyses of the training set were re-used in the analyses for the test set, without modifications. A predicted probability >0.5 resulted in classification as TB case and <0.5 resulted in classification as control. The sensitivity and specificity for the identified signatures was defined by their ability to assign correct probability to participants as being either TB cases or controls. The diagnostic abilities of the signatures in both training and test set were summarized by means of receiver operator characteristics (ROC) curves. Analyses were carried out using R (R Core Team, 2016)^32^ through the interface RStudio (www.rstudio.com).

### Ethics statement

Study A was approved by the Institute Ethics Committee, All India Institute of Medical Sciences (IEC, AIIMS), New Delhi, and the Institutional Ethical Committee, Lady Hardinge Medical College (IEC LHMC), New Delhi, India. The biomarker sub study/experiments were approved by IEC AIIMS on 25.08.2010 and by IEC LHMC on 28.09.2010. Details of study A were registered at clinicaltrials.gov (NCT00801606). Study B, as well as the biomarker experiments were approved by the institutional ethical review board of the St John’s National Academy of Health Sciences, Bangalore, an independent Ethics Committee contracted by Aeras, USA, and the Ministry of Health Screening Committee, Government of India (No. 5/8/9/60/2006-ECD-I dt.10.11.2006). Written informed consent was obtained from parents/guardians, and written assent for participants >7 years. All experiments were performed in accordance with relevant guidelines and regulations.

## Results

### Characteristics of study participants and assignment to training and test set

Of the 133 children included in this study, 36 (27%) had definite intra-thoracic TB disease, 35 (26%) had probable intra-thoracic TB disease, 36 (27%) were asymptomatic HHCs, and 26 (20%) were symptomatic non-TB cases (Fig. 1, Table 1). Off the 66 children with intra-thoracic TB disease from study A, fourteen had additional (cervical) lymph node swelling. Information on potential extra-thoracic manifestations of TB is not available for the 5 TB cases from study B.

Eighty-three participants (62%) were assigned to the training set, comparing gene expression between 47 TB cases (19 definite and 28 probable) and 36 asymptomatic HHCs. Fifty (38%) participants were assigned to the test set, comparing gene expression between 24 TB cases (17 definite and 7 probable) and 26 symptomatic non-TB cases. (Fig. 1, Table 1). Random assignment of TB cases resulted in 40% definite TB cases in the training set, and 70% definite TB cases in the test set.

In the training set, age, sex and BCG-vaccination were similarly distributed among TB cases and asymptomatic HHCs. Whilst all asymptomatic HHCs were exposed to a sibling with TB disease, known TB-exposure was only identified for 16 (34%) of the TB cases. BMI-for-age <2 Z-scores were more frequent in TB cases than in asymptomatic HHCs, 21 (45%) vs 9 (25%). In the test set, the symptomatic non-TB cases were younger than the TB cases, as a result of the symptomatic non-TB cases being selected from a surveillance study of BCG-vaccinated neonates. The proportion of males was higher in the symptomatic non-TB cases, 18 (69%) vs 11 (46%). Whereas no difference was seen for BCG-vaccination, known TB-exposure was more common for TB cases compared to symptomatic non-TB cases, 8 (33%) vs 4 (15%). The signs and symptoms of non-TB cases comprised; weight loss/FTT (23/26, 88%), fever (9/26, 35%) and cough (13/26, 50%), and 6 (23%) had a combination.

### Identification of a 10- and a 7-transcript signature in the training set

The first statistical approach, where age-adjusted LASSO regression analysis was applied directly on the dc-RT MLPA gene expression data, provided a 10-transcript signature, comprising *IFNG*, *NLRP1*, *NLRP3*, *TGFBR2*, *TAGAP*, *NOD2*, *GBP5*, *IFITM1/3*, *KIF1B and TNIP1* (the dc-RT MLPA probes used cannot separate *IFITM1/3*) (Table 2).

**Table 2:** Gene expression and slope coefficients for each biomarker for the identified signatures.

7-transcript signature			10-transcript signature		
Expression TB cases	Gene	Slope Coefficient*	Expression TB cases	Gene	Slope Coefficient*
Increased	**GBP5**	0.32	Increased	**GBP5**	0.36
	**IFITM1/3**	0.74		**IFITM1/3**	0.47
	**KIF1B**	4.26		**KIF1B**	5.28
	MMP9	0.10		NLRP3	10.14
	**NOD2**	0.43		**NOD2**	4.65
	**TNIP1**	12.12		**TNIP1**	4.90
Decreased	CD3E	−2.78	Decreased	IFNG	−30.20
				NLRP1	−1.97
				TAGAP	−0.22
				TGFBR2	−0.47

The 5 genes common for both signatures are denoted in bold-face.

*Slope coefficients are scaled-up by a factor of 10000.

The second approach first applied logistic regression on the dc-RT MLPA data in combination with cross validation, and then age-adjusted LASSO regression analysis was applied on the resulting 24 biomarkers with accuracy ≥0.7. A 7-transcript signature was identified, comprising *MMP9*, *CD3E*, *NOD2*, *GBP5*, *IFITM1/3*, *KIF1B* and *TNIP1*, the latter 5 transcriptomes were common for both signatures (Table 2).

### Performance of the identified biomarker signatures

#### Performance in the training set

**Figure 2:**
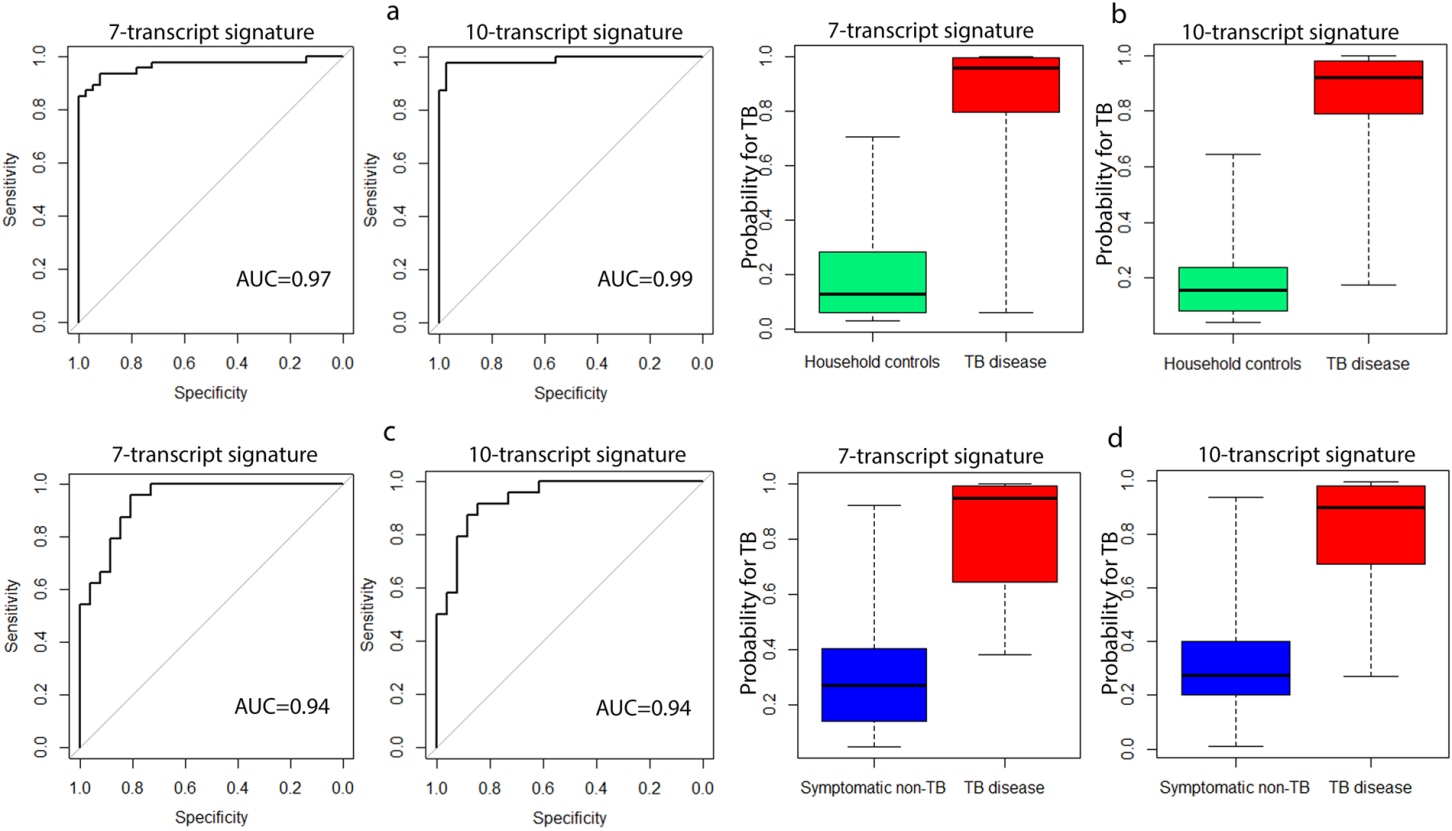
Upper figures: Discriminatory abilities for the identified signatures separating TB cases and asymptomatic HHCs in the training set, shown by: (**a**) receiver operator characteristics (ROC) curves/area under the curve (AUC), and (**b**) box-and-whisker plots (5–95 percentiles). Lower figures: Discriminatory abilities for the identified signatures separating TB cases from symptomatic non-TB cases in the test set, shown by: (**c**) receiver operator characteristics (ROC) curves/area under the curve (AUC), and (**d**) box-and-whisker plots (5–95 percentiles).

The accuracy of the 10-transcript signature for TB disease was very high (AUC 0.99, 95%CI, 0.97–1.00, Fig. 2), correctly classifying 46 of 47 TB cases and 35 of 36 asymptomatic HHCs, corresponding to a sensitivity of 97.9% (95%CI, 87.2–99.9) and a specificity of 97.2% (95%CI, 83.7–99.9). Sensitivity was 100% for definite and 96% for probable TB disease.

The accuracy of the 7-transcript signature for TB disease was also high (AUC 0.97, 95%CI, 0.93–1.00, Fig. 2), correctly classifying 44 of 47 TB cases and 33 of 36 asymptomatic HHCs, corresponding to a sensitivity of 93.6% (95%CI, 81.4–98.3) and a specificity of 91.7% (95%CI, 76.4–97.8). The sensitivity was 94.7% for definite and 92.9% for probable TB disease.

The signatures were further investigated for their capacity to separate TB disease from *Mtb-*infection (TST-positive HHCs). The 7-transcript signature correctly classified 13 of 15 TST-positive HHCs (specificity 86.7%, 95%CI, 58.3–97.7), whereas the 10-transcript signature correctly classified 14 of 15 HHCs (specificity 93.3%, 95%CI, 66.0–99.7).

#### Performance in the test set

The 10-transcript signature provided an AUC of 0.94 (95%CI, 0.88–1.00, Fig. 2), correctly classifying 22 of 24 TB cases and 23 of 26 symptomatic non-TB cases, corresponding to a sensitivity of 91.7% (95%CI, 71.5–98.5) and a specificity of 88.5% (95%CI, 68.7–96.9). Sensitivity was 100% for definite and 71.4% for probable TB disease.

The 7-transcript signature also provided an AUC of 0.94 (95% CI, 0.88–1.00, Fig. 2), correctly classifying 22 of 24 TB cases and 21 of 26 symptomatic non-TB cases, corresponding to a sensitivity of 91.7% (95%CI, 71.5–98.5) and a specificity of 80.8% (95%CI, 60.0–92.7). Sensitivity was 100% for definite and 71.4% for probable TB disease.

The participants misclassified by the 10-transcript signature (3 TB cases, 1 asymptomatic HHC and 3 symptomatic non-TB cases), were also misclassified by the 7-transcript signature.

### Response to anti-TB treatment for the probable TB cases

In the absence of microbiological confirmation, response to anti-TB treatment provides evidence in support of a diagnosis of TB disease. For the 33 probable TB cases from study A, 31 of 33 responded to treatment based on both increased BMI and improvement of CXR findings. The remaining 2 cases also improved, based on one of these two parameters. Notably, children classified with probable TB disease in study A, did not respond to a 7–10-day course of amoxicillin prior to inclusion to the study. For the 2 probable TB cases from study B, no information of treatment response is available.

### Long-term follow-up of asymptomatic HHCs and symptomatic non-TB cases

We were able to trace 33 of the 36 asymptomatic HHCs from study A 3.5 years after study-closeout. Of these 33, 2 developed TB disease; a TST positive child was diagnosed with pulmonary TB one year after participation in the study, and a TST negative child developed extrapulmonary TB 3 years after participation. Both children were classified as not having TB disease by the signatures in the present study.

Regarding the symptomatic non-TB cases (study B), we were able to trace 25 of 26 children, 6 years after study-closeout. Of these 25, none developed TB disease, but one child died in 2016 due to chronic kidney disease.

## Discussion

The importance of effective diagnostics in young children cannot be overemphasized, as *Mtb*-infection is difficult to identify and tends to progress rapidly and often to severe TB disease if left untreated^9^. In the present study, we have applied knowledge derived from a range of previous genome-wide analyses to identify two smaller transcriptional biomarker signatures in WB of Indian children, comprising 7 and 10 transcripts, performing with high diagnostic precision despite the poor nutritional status and young age of many participants. Notably, the signatures meet to a large extent the requirements for a diagnostic POC-test according to the WHO-defined target product profile (TPP):^12^ first, they are non-sputum based and identified in WB. Second, with a sensitivity of 91.7% for all TB cases in the test set, and 100% for definite TB, they perform far better than the proposed ≥66% target for culture confirmed TB. Third, they can potentially differentiate TB disease from *Mtb-*infection. In comparison, the Xpert MTB/RIF has a sensitivity of 68.8% for culture confirmed TB when performed on gastric lavage^4^. However, given the paucibacillary nature of pediatric TB disease, culture of gastric lavage/induced sputum misses true disease in about 70% of cases, suggesting that the sensitivity of the Xpert MTB/RIF might be as low as 21% in this group^4^. The identified signatures could provide a major improvement with regards to sensitivity and availability of samples (WB), if translated to a POC-test, possibly by using technology similar to the Xpert MTB/RIF. The 7- and 10-transcript signature provided a specificity in the test set of 80.8% and 88.5%, respectively. This does not reach the recommended specificity of ≥98% for a POC-test^12^, but might be further improved by including additional or alternate genes.

Whereas most gene expression studies are performed in adults in Africa, the present study is conducted in children in India, thereby providing novel knowledge, as gene expression is likely to differ with regard to age, as well as genetic and environmental background^15, 28^. In this regard, Verhagen *et al*. found that in adults from South Africa, Gambia and the United Kingdom, their signature discriminated TB disease from latent TB, but the signatures identified in these cohorts, did not have the same ability in their own pediatric cohort^15, 17, 22, 23^. This indicates that transcriptional signatures identified in adults cannot always be extrapolated to children. Notably, the signature dominated by type- I interferon-inducible genes identified in adults by Berry *et al*.^17^ had poor discriminatory power in the Warao-Amerindian children^15^, whereas the findings by Anderson *et al*. supported up-regulation of these genes in children with TB disease^14^. In the present study, 2 of the 5 genes common between the two signatures, i.e. *GBP5 and IFITM1/3*, supports the importance of type-1 interferon signaling in pediatric TB disease. *GBP5* was also present in the signatures identified by Anderson *et al*.^14^, and part of the 3-gene signature identified in the comprehensive multicohort analysis by Sweeney *et al*.^16^, as the gene-combination most predictive of TB disease. In children, the mean sensitivity achieved by this 3-gene combination was 86% for culture confirmed TB vs. latent TB. This highlights the performance of the signatures for definite TB in our study.

In studies of this kind, it is not possible to be certain of the identity of the TB index case, or to determine if TST-positive HHCs are recently or long-term infected. These different states could affect their expression profiles. However, given the combination of their young age and documented exposure to a sibling with TB, we consider recent infection to be the most plausible explanation. To gain some insight into outcomes, 33 of the 36 HHCs were traced 3.5 years after study-closeout. Only 2 of these developed TB disease; a TST positive child developed pulmonary TB one year after study participation, and a TST negative child developed extrapulmonary TB 3 years after participation. Both children were classified as not having TB by the signatures. Though the sample size is small, this suggests that the biosignatures identify TB disease, and not asymptomatic TB infection.

The relatively small number of TB cases and controls, particularly in the test set, represent a limitation to the study, warranting exploration and validation in other, larger cohorts. Moreover, the signatures do not meet the WHO recommendations for a POC-test with regards to specificity, and the fact that steps towards such a test still remain, represents a rationale for presenting both signatures in the present exploratory study. The 10-transcript signature yielded somewhat higher diagnostic precision, and includes the highest number of genes, all possible candidates for a future POC-test signature. The strength of the 7-transcript signature lies in the initial logistic regression step, reducing possibilities for overfitting. The consistency of having 5 genes in common strengthens the relevance of both signatures. Future exploration could include the investigation of performance for all 12 biomarkers combined, but this will warrant alternate or modified statistical approaches. Partly because our previous studies were limited to a panel of 48 biomarkers, and also due to some overlap of the study subjects selected for the studies, we consider future exploration of the present expanded gene panel more informative and stringent, than testing the performance of previously identified biomarkers signatures in the current study.

Nevertheless, the novel signatures support findings of genes with relevance to TB-pathology identified in our recent biomarker signature study. In this study, *CD3E* and *TGFBR2* were part of a biosignature separating TB cases from asymptomatic HHCs, and *MMP9* was significantly upregulated in TB cases^24^. This study also showed that differences in biomarkers signatures were most apparent between the most polar clinical groups of the TB disease spectrum, corresponding to 100% sensitivity for the signatures for definite TB, in the test set of the present study. We acknowledge that randomization of TB cases resulted in 40% definite TB in the training set vs. 70% in the test set, but we judged the total number of TB cases and size of each group to be of greater importance, than a 50/50 distribution of definite TB cases between the training and the test set.

Further, despite adjustment for age in all steps of the analyses, we cannot rule out residual confounding; that the ability of the signatures to separate TB cases from symptomatic non-TB cases in the test set is not related only to the absence or presence of TB disease, but might be influenced by age-dependent gene-expression, as the mean ages differed between the groups. However, the 10-transcript signature correctly classified the 5 of 6 TB cases <2 years, and the 7-transcript signature correctly classified 4 of 6. Although our numbers are too small for definite conclusion in this particular age group, and exploration in larger populations is needed, these findings speak in favor of the age-independent, discriminatory abilities for TB disease of the identified signatures.

With regards to confounding, underweight is a feature well recognized to contribute to TB, but also to be a result of this disease. The design of our study does not enable us to decide whether the signatures are influenced by nutrition status, but accurate separation of TB cases from controls in both training and test set, regardless of the unequally distributed BMI between the control groups, speaks against nutrition status as an important confounder. Further, both control groups contain TST positive and negative individuals. Although this lack of uniformity could be viewed as a limitation, this is indeed reflective of real-life clinical practice, where a POC-test must have the ability of separating TB cases from other children seeking health-care, regardless of their TST-status.

In conclusion, we have applied knowledge gained from multiple genome-wide analyses, by targeting promising genes identified in these studies, using the novel, accurate and inexpensive dcRT-MLPA, which provides a platform more easily transferable to a future POC-test. We have identified small, consistent biomarker signatures with solid abilities to discriminate TB cases from asymptomatic HHCs. Further, in the study cohort, these signatures also proved to be powerful tools in discriminating TB cases from children with symptoms from other causes, reflective of the situation encountered in real-life clinical practice. We have confirmed findings of others in an Indian population that differs with respect to age, genetic background and environment. Altogether, this study provides additional insight into gene expression in pediatric TB disease, and contributes to optimism for future non-sputum based POC-diagnostic tools for pediatric TB.

### Data availability statement

The datasets generated during and/or analysed during the current study, are available from the corresponding authors on request.

## Electronic supplementary material

Supplementary Table S1
