# Supplementary material for: Novel transcriptional signatures for sputum-independent diagnostics of tuberculosis in children
Source: Sci Rep. 2017 Jul 19;7:5839. doi: 10.1038/s41598-017-05057-x (PMC5517635; doi:10.1038/s41598-017-05057-x)
Supplement: Supplementary file 1 — Supplementary Table S1 [file 41598_2017_5057_MOESM1_ESM.pdf]

Supplementary information

"Novel transcriptional signatures for sputum-independent diagnostics of tuberculosis in children"

Authors: John Espen Gjøen[1], Synne Jenum*[1,2], Dhanasekaran Sivakumaran*[1], Aparna Mukherjee[3], Macaden R[4], Sushil K. Kabra[3], Rakesh Lodha[3], Tom HM Ottenhoff[5], Marielle C Haks[5], Timothy Mark Doherty[6], Christian Ritz#[7] and Harleen M.S. Grewal# [1,8]

Authors' Addresses: [1]Department of Clinical Science, Faculty of Medicine and Dentistry, University of Bergen, Bergen, Norway. [2]Department of Infectious Diseases, Oslo University Hospital, Oslo, Norway. [3]Department of Pediatrics, All India Institute of Medical Sciences, New Delhi, India. [4]Division of Infectious Diseases, St. John's Research Institute, Koramangala, Bangalore, India. [5]Department of Infectious Diseases Group, Immunology and Immunogenetics of Bacterial Infectious Disease, Leiden University Medical Center, Leiden, the Netherlands. [6]GlaxoSmithKline Vaccines, Wavre, Belgium. [7]Department of Nutrition, Exercise and Sports, University of Copenhagen, Copenhagen, Denmark. [8]Department of Microbiology, Haukeland University Hospital, University of Bergen, Bergen, Norway.

***Equal contribution**

# Corresponding authors

Corresponding authors:

Harleen M.S Grewal MD PhD

Professor and Senior Consultant,
Department of Clinical Science, Faculty of Medicine and Dentistry
University of Bergen & Department of Microbiology
Haukeland University Hospital
Bergen 5021, Norway
Phone: +47 55-97-4631; Mobile: +47 99450554
Email: Harleen.Grewal@.uib.no

Christian Ritz PhD

Associate Professor

Department of Nutrition, Exercise and Sports, University of Copenhagen,

Copenhagen, Denmark

Phone: +45 35 33 25 36

Email: ritz@nexs.ku.dk.

Supplementary Table S1: The genes and the gene names of the 145 unique genes included in the study, and their distribution in 3 gene panels. Repeated genes (genes present in more than one panel), are only listed once.

Gene	Gene Name
	**Panel A[1,2]**
**ABR**	Active BCR-Related gene
**BCL2**	B-Cell CLL/Lymphoma 2
**BLR1**	Burkitt Lymphoma Receptor 1
**CASP8**	Cysteine-Aspartic Acid Protease 8
**CCL13**	C-C Motif Chemokine Ligand 13
**CCL19**	C-C Motif Chemokine Ligand 19
**CD4**	CD4 Molecule
**CD14**	CD14 Molecule
**CD19**	CD19 Molecule
**CD163**	CD163 Molecule
**CTLA4**	Cytotoxic T-lymphocyte Antigen 4
**FASLG**	Fas Ligand
**FOXP3**	Forkhead Box P3
**FPR1**	Formyl Peptide Receptor 1
**IL4**	Interleukin 4
**IL4d2**	Interleukin 4 Delta 2
**IL7R**	Interleukin 7 Receptor
**LTF**	Lacto-transferrin
**MARCO**	Macrophage Receptor
**MMP9**	Matrix Metallo-Peptidase 9
**NCAM1**	Neural Cell Adhesion Molecule 1
**RAB13**	Ras Related Protein-13
**RAB24**	Ras Related Protein-24
**RAB33A**	Ras Related Protein-33A
**SEC14L1**	SEC14 Cytosolic Factor Family
**SPP1**	Secreted Phosphoprotein 1
**TGFBR2**	Transforming Growth Factor Beta Receptor 2
**TIMP2**	Tissue Inhibitor of Metallo-proteinases 2
**TNFRSF1A**	TNF Receptor Superfamily Member 1A

**TNFRSF1B**	TNF Receptor Superfamily Member 1B
	**Panel B[3]**
**AIRE**	Autoimmune regulator
**AREG**	Amphiregulin
**BPI**	Bactericidal/Permeability-Increasing Protein
**CAMTA1**	Calmodulin Binding Transcription Activator 1
**CCL2**	C-C Motif Chemokine Ligand 2
**CCL22**	C-C Motif Chemokine Ligand 22
**CCL4**	C-C Motif Chemokine Ligand 4
**CCL5**	C-C Motif Chemokine Ligand 5
**CCR7**	C-C Motif Chemokine Receptor 7
**CD209**	CD209 Molecule
**CD3E**	CD3e Molecule
**CD8A**	CD8a Molecule
**CLEC7A**	C-Type Lectin Domain Family 7 Member A
**CXCL13**	C-X-C Motif Chemokine Ligand 13
**FCGR1A**	Fc Fragment Of IgG Receptor Ia
**FLCN1**	Folliculin
**GATA3**	GATA Binding Protein 3
**GNLY**	Granulysin
**GZMA**	Granzyme A
**GZMB**	Granzyme B
**IFNG**	Interferon Gamma
**IL2**	Interleukin2
**IL5**	Interleukin 5
**IL9**	Interleukin 9
**IL10**	Interleukin 10
**IL13**	Interleukin 13
**IL15**	Interleukin 15
**IL12A**	Interleukin 12A
**IL17A**	Interleukin 17A
**IL23A**	Interleukin 23A
**IL1B**	Interleukin 1B

**IL12B**	Interleukin 12B
**LAG3**	Lymphocyte Activating 3
**MRC1**	Mannose Receptor C-Type 1
**MRC2**	Mannose Receptor C-Type 2
**NEDD4L**	Neural Precursor Cell Expressed, Developmentally Down-Regulated 4-Like, E3 Ubiquitin Protein Ligase
**NLRC4**	NLR Family CARD Domain Containing 4
**NLRP1**	NLR Family Pyrin Domain Containing 1
**NLRP2**	NLR Family Pyrin Domain Containing 2
**NLRP3**	NLR Family Pyrin Domain Containing 3
**NLRP4**	NLR Family Pyrin Domain Containing 4
**NLRP6**	NLR Family Pyrin Domain Containing 6
**NLRP7**	NLR Family Pyrin Domain Containing 7
**NLRP10**	NLR Family Pyrin Domain Containing 10
**NLRP11**	NLR Family Pyrin Domain Containing 11
**NLRP12**	NLR Family Pyrin Domain Containing 12
**NLRP13**	NLR Family Pyrin Domain Containing 13
**NOD1**	Nucleotide Binding Oligomerization Domain Containing 1
**NOD2**	Nucleotide Binding Oligomerization Domain Containing 2
**PRF1**	Perforin 1
**PTPRCv1**	Protein tyrosine phosphatase receptor type Cv1
**PTPRCv2**	protein tyrosine phosphatase receptor type Cv2
**RORC**	RAR Related Orphan Receptor C
**TAGAP**	T-Cell Activation RhoGTPase Activating Protein
**TBC1D7**	TBC1 Domain Family Member 7
**TBX21**	T-Box 21
**TGFB1**	Transforming Growth Factor Beta 1
**TLR1**	Toll Like Receptor 1
**TLR2**	Toll Like Receptor 2
**TLR3**	Toll Like Receptor 3
**TLR4**	Toll Like Receptor 4
**TLR5**	Toll Like Receptor 5
**TLR6**	Toll Like Receptor 6
**TLR7**	Toll Like Receptor 7

**TLR8**	Toll Like Receptor 8
**TLR9**	Toll Like Receptor 9
**TLR10**	Toll Like Receptor 10
**TNF**	Tumor Necrosis Factor
**TNFRSF18**	TNF Receptor Superfamily Member 18
**TWIST1**	Twist Family BHLH Transcription Factor 1
**ZNF331**	Zinc Finger Protein 331
**ZNF532**	Zinc Finger Protein 532
	**Panel C[4,5]**
**ASAP1**	ArfGAP With SH3 Domain, Ankyrin Repeat And PH Domain 1
**B2M**	Beta-2-Microglobulin
**BMP6**	Bone Morphogenetic Protein 6
**CCL11**	C-X-C Motif Chemokine Ligand 11
**CCL3**	C-C Motif Chemokine Ligand 3
**CD274**	CD274 Molecule
**CX3CL1**	C-X3-C Motif Chemokine Ligand 1
**CXCL9**	C-X-C Motif Chemokine Ligand 9
**CXCL10**	C-X-C Motif Chemokine Ligand 10
**DSE**	Dermatan Sulfate Epimerase
**EGF**	Epidermal Growth Factor
**GBP1**	Guanylate Binding Protein 1
**GBP2**	Guanylate Binding Protein 2
**GBP5**	Guanylate Binding Protein 5
**GUSB**	Glucuronidase Beta
**HCK**	HCK Proto-Oncogene, Src Family Tyrosine Kinase
**HPRT**	Hypoxanthine Phosphoribosyltransferase 1
**IFI6**	Interferon Alpha Inducible Protein 6
**IFI16**	Interferon Gamma Inducible Protein 16
**IFI35**	Interferon Induced Protein 35
**IFI44**	Interferon Induced Protein 44
**IFI44L**	Interferon Induced Protein 44 Like
**IFIH1**	Interferon Induced With Helicase C Domain 1
**IFIT2**	Interferon Induced Protein With Tetratricopeptide Repeats 2

**IFIT3**	Interferon Induced Protein With Tetratricopeptide Repeats 3
**IFIT5**	Interferon Induced Protein With Tetratricopeptide Repeats 5
**IFITM3**	Interferon Induced Transmembrane Protein 3
**IL6**	Interleukin 6
**INDO**	Indoleamine 2,3-Dioxygenase 1
**IRF7**	Interferon Regulatory Factor 7
**KIF1B**	Kinesin Family Member 1B
**LYN**	LYN Proto-Oncogene, Src Family Tyrosine Kinase
**OAS1**	2'-5'-Oligoadenylate Synthetase 1
**OAS2**	2'-5'-Oligoadenylate Synthetase 2
**OAS3**	2'-5'-Oligoadenylate Synthetase 3
**SLAMF7**	SLAM Family Member 7
**SOCS1**	Suppressor Of Cytokine Signaling 1
**STAT1**	Signal Transducer And Activator Of Transcription 1
**STAT2**	Signal Transducer And Activator Of Transcription 2
**TAP1**	Transporter 1, ATP Binding Cassette Subfamily B Member
**TAP2**	Transporter 2, ATP Binding Cassette Subfamily B Member
**TNIP1**	TNFAIP3 Interacting Protein 1
**VEGF**	Vascular Endothelial Growth Factor A

References:

1. Joosten, S. A. *et al.* Identification of biomarkers for tuberculosis disease using a novel dual-color RT-MLPA assay. *Genes Immun* 13, 71-82, doi:10.1038/gene.2011.64 (2012)

2. Dhanasekaran, S. *et al.* Identification of biomarkers for *Mycobacterium tuberculosis* infection and disease in BCG-vaccinated young children in Southern India. *Genes Immun* 14, 356-364, doi:10.1038/gene.2013.26 (2013)

3. Joosten, S. A. *et al* . A Helicopter Perspective on TB Biomarkers: Pathway and Process Based Analysis of Gene Expression Data Provides New Insight into TB Pathogenesis. PLoS ONE 8(9): e73230 (2013)

4. Berry, M. P. *et al* . An interferon-inducible neutrophil-driven blood transcriptional signature in human tuberculosis. Nature 466, 973-977, doi:10.1038/nature09247 (2010)

5. Fletcher, H. A. *et al* . Human newborn bacille Calmette-Guerin vaccination and risk of tuberculosis disease: a case-control study. BMC medicine 14, 76, doi:10.1186/s12916-016-0617-3 (2016)
